# The nutraceutical benefits of subfractions of *Abelmoschus esculentus* in treating type 2 diabetes mellitus

**DOI:** 10.1371/journal.pone.0189065

**Published:** 2017-12-07

**Authors:** Chien-Ning Huang, Chau-Jong Wang, Chih-Li Lin, Hui-Ting Lin, Chiung-Huei Peng

**Affiliations:** 1 Department of Internal Medicine, Chung-Shan Medical University Hospital, Taichung, Taiwan; 2 Institute of Medicine, Chung-Shan Medical University, Taichung, Taiwan; 3 Institute of Biochemistry, Microbiology and Immunology, Chung-Shan Medical University, Taichung, Taiwan; 4 Division of Basic Medical Science, Hungkuang University, Shalu District, Taichung City, Taiwan; Institute of Biochemistry and Biotechnology, TAIWAN

## Abstract

*Abelmoschus esculentus* (AE), a commonly consumed vegetable, is well-known for its anti-hyperglycemic effects. However, few scientific reports have identified its targets because mucilage increases the difficulty of manipulation. We recently reported extraction steps to obtain subfractions of AE, which were found to attenuate the adverse effects of high glucose and fatty acid *in vitro*. In this study, we used modified extraction steps and type 2 diabetic rats to explore whether AE subfractions can improve the metabolic disturbances caused by insulin resistance *in vivo*. AE subfractions (F1, F2, and FR) were prepared. The type 2 diabetes model was induced by feeding male Sprague-Dawley rats with a high-fat diet and injecting them with 35 mg/kgbw streptozotocin when their body weight reached 475 ± 15 g. After a hyperglycemic status had been confirmed, the rats were tube-fed with or without different doses of AE subfractions. Serum glucose, lipid markers, insulin, HbA1c and HOMA-IR were measured in the following 12 weeks. Serum glucose promptly increased and insulin resistance was noted in the diabetic rats (glucose: 360–500 mg/dl, HOMA-IR 9.8–13.8). F2, rich in polysaccharides and carbohydrates, was most effective in attenuating hyperglycemia and insulin resistance (glucose: 200 mg/dl; HOMA-IR: 5.3) and especially HbA1C (from 8.0% to 6.5%). All of the AE subfractions lowered the level of triglycerides and free fatty acid, but not the level of total cholesterol. FR significantly increased the high-density lipoprotein/low-density lipoprotein ratio, indicating its benefits for lipoprotein profiles. While F2 and FR were associated with weight gain, F1 possessed an anti-obese effect. In conclusion, whether it is consumed as a vegetable or as a nutraceutical, AE has the potential to be an adjuvant therapy for diabetes. AE subfractions could be developed individually and deserve further investigation.

## Introduction

Diabetes mellitus is a highly prevalent disease worldwide and is associated with increased rates of morbidity and mortality. Diabetes-associated metabolic disorders manifest as hyperglycemia and increased advanced glycated hemoglobin (HbA1c) through disturbances of insulin secretion or action efficiency, and these disorders play a central role in diabetes clinical care and pathophysiology. In addition, dyslipidemia with high levels of triglycerides (TG), cholesterol, low-density lipoprotein (LDL) and a low level of high-density lipoprotein (HDL) are frequently noted in patients with diabetes. Type 2 diabetes is the most prevalent form of diabetes. Obesity is known to be an important morbidity factor in the pathogenesis of type 2 diabetes, and it is associated with an overproduction of free fatty acid (FFA) [1].

*Abelmoschus esculentus* (AE; also known as okra) is one flowering plant of the mallow family [2]. The fruit of AE is consumed as a popular vegetable in many countries. In addition to its high fiber, vitamin, and trace element contents [3], AE is also known for its medicinal value, especially with regards to an anti-hyperglycemic effect. Although AE is generally viewed as being advantageous for diabetic patients, few scientific reports have identified the clinical targets that AE acts on. A previous work of Sabitha et al. revealed that AE reduced blood glucose and lipids, and increased body weight in streptozotocin (STZ)-induced diabetic rats [4]. Possessing a good anti-oxidation ability, AE has been shown to decrease lipid peroxidation, increase the levels of superoxide dismutase, catalase, and glutathione peroxidase, and the reduced glutathione in the liver, kidney and pancreas of diabetic rats [5]. However, in these reports, the experimental animals were fed with AE powder of the seeds and peel which was crude, preventing the bioactive components from being identified. In fact, AE contains abundant mucilage which increases the difficulty in isolation, analysis and further tests with bio-models.

Our previous report successfully demonstrated extraction steps and obtained a series of subfractions from AE which were analyzed for their chemical composition, and tested for their individual effects and molecular targets to prevent diabetic renal epithelial to mesenchymal transition [6]. In addition, we recently demonstrated that AE subfractions can prevent FFA-induced β cell apoptosis by inhibiting dipeptidyl peptidase-4, an important target of type 2 diabetes therapy [7]. Based on this, in the present study, we used modified extraction steps and tested AE subfractions on type 2 diabetic rats with insulin resistance [8, 9]. We aimed to explore whether AE subfractions can improve the metabolic disturbances caused by insulin resistance.

## Materials and methods

### Preparation of AE subfractions and chemical analysis

**Fig 1:**
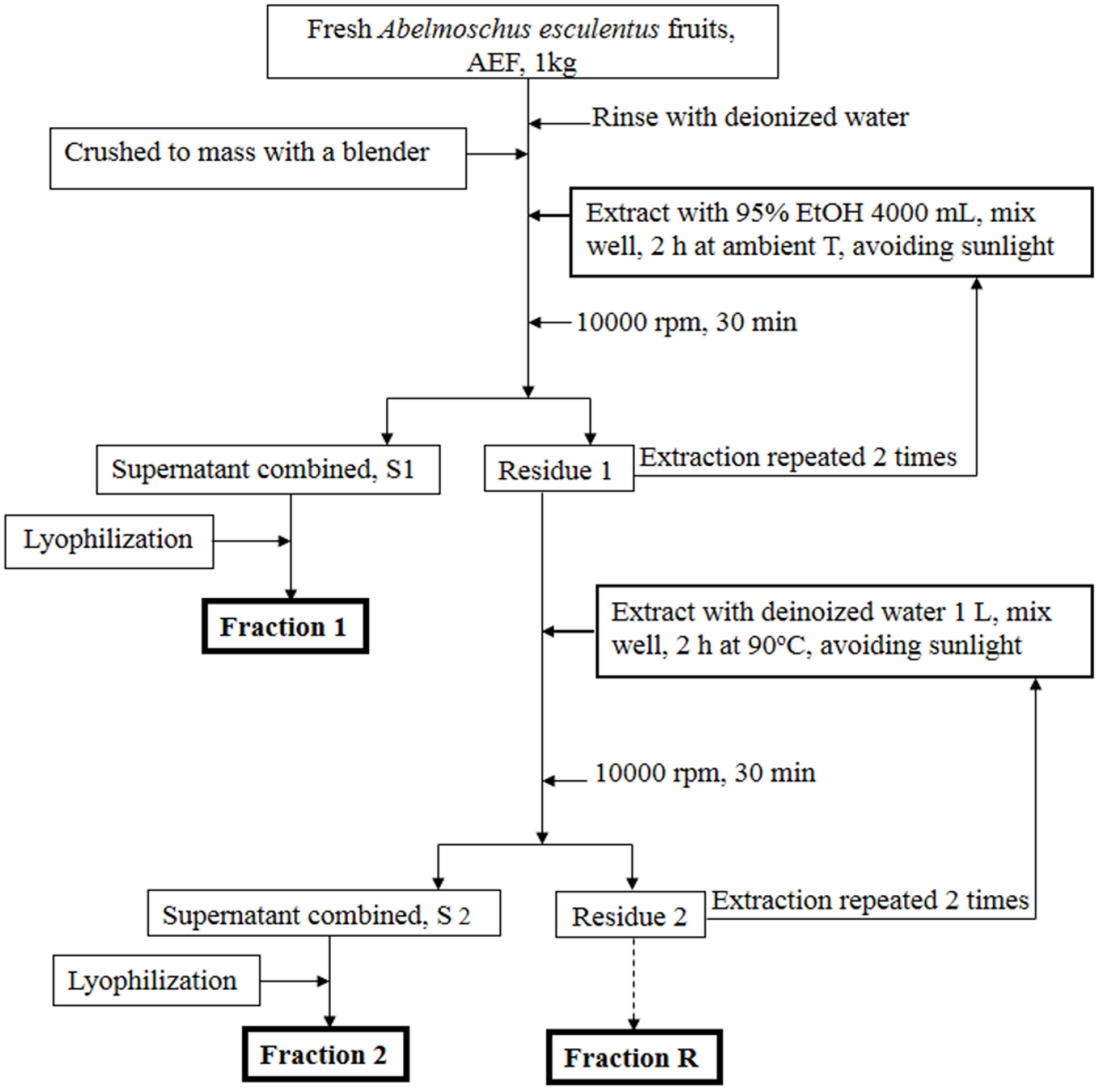
The procedures for the extraction of AE subfractions.

AE was purchased from Chuchi (Chiayi, Taiwan). The subfractions of AE (F1, F2 and the residue FR) were prepared according to the procedures shown in Fig 1. The yields of dry base of F1, F2 and FR were 1.08%, 12.59%, and 48.27%, respectively. F1, the alcohol-extracted subfraction of AE, was previously analyzed using HPLC and LC-MS/MS (6). F1 was composed of at least 10 compounds, including quercetin glucosides and pentacyclic triterpene ester [S1 Table]. The F2 portion of AE contained a large amount of carbohydrates and polysaccharides. Monosaccharide analysis and uronic determination revealed that F2 was rich in uronic acid (23.14%), galactose (18.92%), glucose (18.26%) and myo-inositol (14.21%) [S2 Table]; rhamnose, glucosamine, and fucose were also found to be quite abundant. Using GPC analysis, the mean molecular weight of F2 was estimated to be 671 kDa (6).

### Animal experiments

The animal experimental project was approved by the Animal Model Experimental Ethics Committee of Chung-Shan Medical University, and was conducted in accordance with the recommendation of the Guide for the Care and Use of Laboratory Animals of the National Institutes of Health. Briefly, male Sprague-Dawley rats (weight 250±20 g, age 7 weeks) were obtained from LuxBiotech Co., Taiwan. The rats, 8 in each group and 4 in each cage, were acclimated and fed basic chow consisting of 12% fat for the first week before the experiments. The animal room was maintained at a 12 h light/dark cycle, 25°C, and 55±5% relative humidity. All animals had free access to food and water. The protocol described by Yang et al (8) was used to induce type 2 diabetes in the rats. Using the formulation described in AIN-76, normal and high-fat diets (HFD) were prepared and rationed according to the formula previously reported [S3 Table]. After 8 weeks, when the average body weight was 475 ±15 g, the HFD-fed rats were injected intraperitoneally (ip) with 35 mg/kgbw of STZ. The other rats received only the same amount of 0.1 M citric acid buffer (pH 4.5). About 2 weeks later, when the hyperglycemic status was confirmed, the rats were tube-fed with or without different doses of AE subfractions. Briefly, the rats were divided into the following groups: control (normal diet), C1-C3 (normal diet with 0.45 mg/kg F1, F2, or FR added), HFD + STZ (HFD with STZ injection; diabetes model), HFD + STZ + F1 (L) (diabetes with 0.23 mg/kg F1), HFD + STZ + F1 (H) (diabetes with 0.45 mg/kg F1), HFD + STZ + F2 (L) (diabetes with 0.23 mg/kg F2), HFD + STZ + F2 (H) (diabetes with 0.45 mg/kg F2), HFD + STZ + FR (L) (diabetes with 0.23 mg/kg FR), and HFD + STZ + FR (H) (diabetes with 0.45 mg/kg F3). The body weight, serum glucose and insulin were measured every 2 weeks. At the end of the experiment, the animals were sacrificed under 4–8 psi of CO_2_. Although unconsciousness often occurred within 1 minute, the rats were left in the container for at least 3–5 minutes to ensure death, which was verified no cardiac pulse.

### Serum biochemical assays

All animals were fasted overnight for 8–10 h before the experiments were conducted.

Serum samples were collected into EDTA tubes and centrifuged at 3,000 rpm for 10 min at 4°C. Concentrations of TG, total cholesterol, LDL, HDL, and FFA, BUN and GOT were measured by enzymatic colorimetric methods using commercial kits (Randox Laboratories Ltd., Antrim, U.K.). Plasma glucose was measured by enzymatic colorimetric methods using an automatic analyzer (Olympus AU2700, Olympus Co., Tokyo, Japan). Plasma insulin concentration was determined by ELISA using a rat insulin enzyme immunoassay kit (Mercodia, Uppsala, Sweden).

### HbA1C measurement

To measure the level of HbA1c, an inhibition of latex agglutination assay was used (HbA1c-specific mouse monoclonal antibody adsorbent onto latex particles, DCA Vantage Analyzer, Siemens Healthcare Diagnostics, Deerfield, IL, USA). A synthetic polymer containing multiple copies of the immunoreactive portion of HbA1c caused agglutination of latex coated with HbA1c-specific mouse monoclonal antibodies. The agglutination reaction increased scattering of light, which was measured as an increased absorbance at 531 nm. HbA1c in whole blood specimens competed for the limited number of antibody latex binding sites, thus inhibiting agglutination and decreasing the absorbance. The HbA1c concentration was then quantified using a calibration curve of absorbance versus HbA1c concentration. The percent of HbA1c in the sample was then calculated as: %HbA1c = [HbA1c] / [total hemoglobin] *×* 100

### Homeostasis model of assessment for insulin resistance index (HOMA-IR) calculation

HOMA-IR was calculated as: HOMA-IR = [glucose (mmol/L) × insulin (μU/mL)/22.5], using fasting values [10].

### Statistical analysis

The statistical software SPSS version 12.0 was used to analyze the data. One-way ANOVA was performed (p < 0.05), with Bonferroni’s multiple comparison post-test.

## Results

### AE subfractions reduced hyperglycemia and attenuated insulin resistance

Compared with the control group, the baseline serum glucose levels promptly increased in the type 2 diabetic rats, from a baseline of 80 mg/dl to a high of 360–500 mg/dl. Four weeks later, F2 (H) was the first subfraction to show an anti-hyperglycemic effect. F2 (H) continued to decrease serum glucose until the end of the experiments. F1 (H) also decreased serum glucose at 6, 8 and 10 weeks, but not as effectively as F2 (H). Among all of the subfractions, FR had the least potent anti-hyperglycemic effect, with an effect only at 8 and 10 weeks (Table 1).

**Table 1:** Effect of AE subfractions on the level of serum glucose.

mg/dL	week						
	0	2	4	6	8	10	12
**Control**	83.50±4.33	94.0±4.14	90.75±5.25	83.75±5.44	86.50±3.97	75.75±2.29	77.25±2.95
**HFD+STZ**	373.00±47.12^###^	358.13±22.13^###^	396.00±32.31^###^	444.14±17.77^###^	472.14±19.24^###^	533.29±19.54^###^	445.25±23.45^###^
**H+S+F1(L)**	357.00±17.40^###^	378.75±16.13^###^	401.67±58.99	419.75±19.66	458.00±19.03	469.67±50.60	404.00±34.49
**H+S+F1(H)**	412.50±22.22^###^	391.3±45.10^###^	375.67±25.09	387.40±26.31*	394.83±15.80*	447.33±23.68*	396.40±16.62
**H+S+F2(L)**	501.20±43.94^###^	471.60±34.01^###^	477.33±34.90	440.50±36.80	379.00±32.25*	441.50±11.65**	419.00±33.97
**H+S+F2(H)**	442.83±44.71^###^	480.80±33.66^###^	319.60±16.15*	315.00±57.76*	260.33±16.86**	256.00±32.39**	209.33±46.05**
**H+S+FR(L)**	381.40±29.11^###^	479.75±43.25^###^	471.25±36.22	421.67±20.17	422.75±18.05	442.67±35.41*	429.00±22.00
**H+S+FR(H)**	397.00±12.16^###^	465.40±36.62^###^	440.33±32.53	448.67±40.17	366.10±23.16*	418.00±18.00**	411.50±45.50

Note: control (normal diet), HFD + STZ (diabetes model), HFD + STZ +F1 (L) (diabetes with 0.23 mg/kg F1), HFD + STZ +F1 (H) (diabetes with 0.45 mg/kg F1), HFD + STZ +F2 (L) (diabetes with 0.23 mg/kg F2), HFD + STZ +F2 (H) (diabetes with 0.45 mg/kg F2), HFD + STZ +FR (L) (diabetes with 0.23 mg/kg FR), and HFD + STZ +FR (H) (diabetes with 0.45 mg/kg F3). Data were statistically analyzed with ANOVA.

^###^p<0:001 compared with the control.

*p<0:05

**p<0:01 compared with the HFD + STZ group. From 4th wk to 12wk, only the effective AE-treated groups would be marked.

The dynamic change of serum insulin level is shown in Table 2.

**Table 2:** Effect of AE subfractions on the level of plasma insulin.

mU/L	week						
	0	2	4	6	8	10	12
**Control**	13.01±0.11	13.80±0.27	13.41±0.13	11.71±0.70	12.40±0.62	12.15±0.73	11.84±0.47
**HFD+STZ**	13.07±0.18	12.36±0.24	11.57±0.78	11.59±0.58	10.76±0.58	8.94±0.13	8.58±0.15
**H+S+F1(L)**	11.65±0.25	11.84±0.23	9.76±0.24	7.32±0.22	8.24±0.25	8.52±0.12	8.38±0.39
**H+S+F1(H)**	13.53±0.39	14.16±0.14	12.93±0.87	6.15±0.22	6.47±0.13	9.83±0.29	6.69±0.78
**H+S+F2(L)**	11.00±0.27	13.83±0.41	12.03±0.90	10.42±0.97	7.24±0.14	10.32±0.62	8.70±0.84
**H+S+F2(H)**	9.03±0.21	12.14±0.36	10.71±0.18	8.28±0.25	8.22±0.08	7.79±0.39	9.85±0.35
**H+S+FR(L)**	11.84±0.25	12.46±0.24	11.05±0.44	6.89±0.34	7.45±0.19	7.95±0.64	7.54±0.45
**H+S+FR(H)**	11.50±0.37	11.94±0.11	10.69±0.40	6.22±0.26	6.24±0.12	9.05±0.14	6.94±0.06

The insulin secretion of the diabetic rats continued until 8 weeks and decreased thereafter. Histological findings revealed that AE treatment prevented the exacerbation of β islets [S1 Fig].

**Fig 2:**
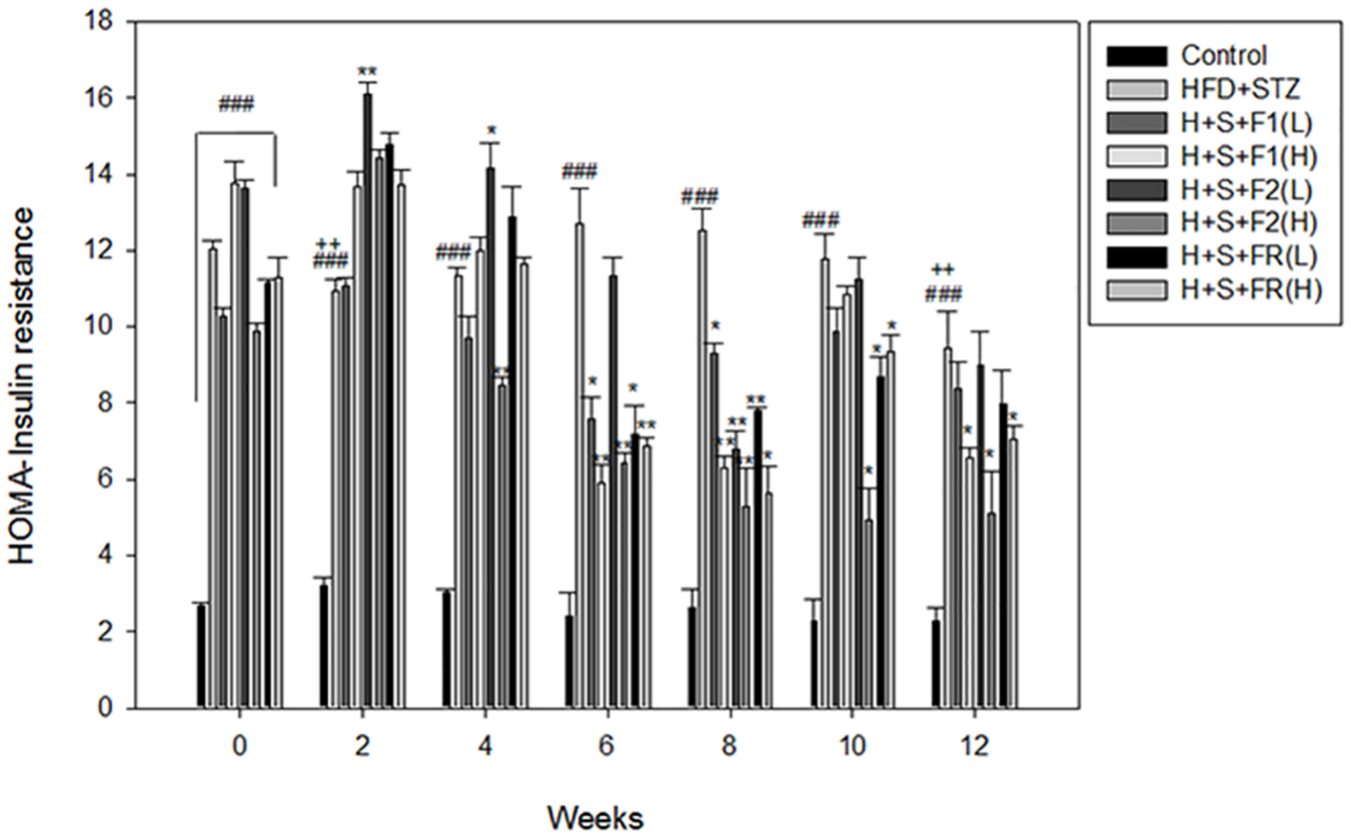
Effect of AE on hyperglycemia and insulin resistance in type 2 diabetic rats. Serum from the experimental animals was collected. The glucose and insulin concentrations were analyzed, and HOMA-IR was calculated. Data are presented as mean±SD (n = 8 per group), and analyzed with ANOVA and post- test. ###*p* < 0.001, compared with the controls; *p < 0.05, **p < 0.01, compared with HFD+STZ; ++*p* < 0.01, compared with the same group at 0 week.

All of the diabetic rats had insulin resistance in the first 2 weeks. HOMA-IR ranged from 9.8–13.8 vs. 2.8 (the controls). Among all of the subfractions, F2 (H) was superior in decreasing insulin resistance at 4–12 weeks. F1 and FR were also effective in attenuating insulin resistance, showing an effect from 6 weeks to the end of the experiments (Fig 2).

### AE subfractions decreased the formation of HbA1C

**Fig 3:**
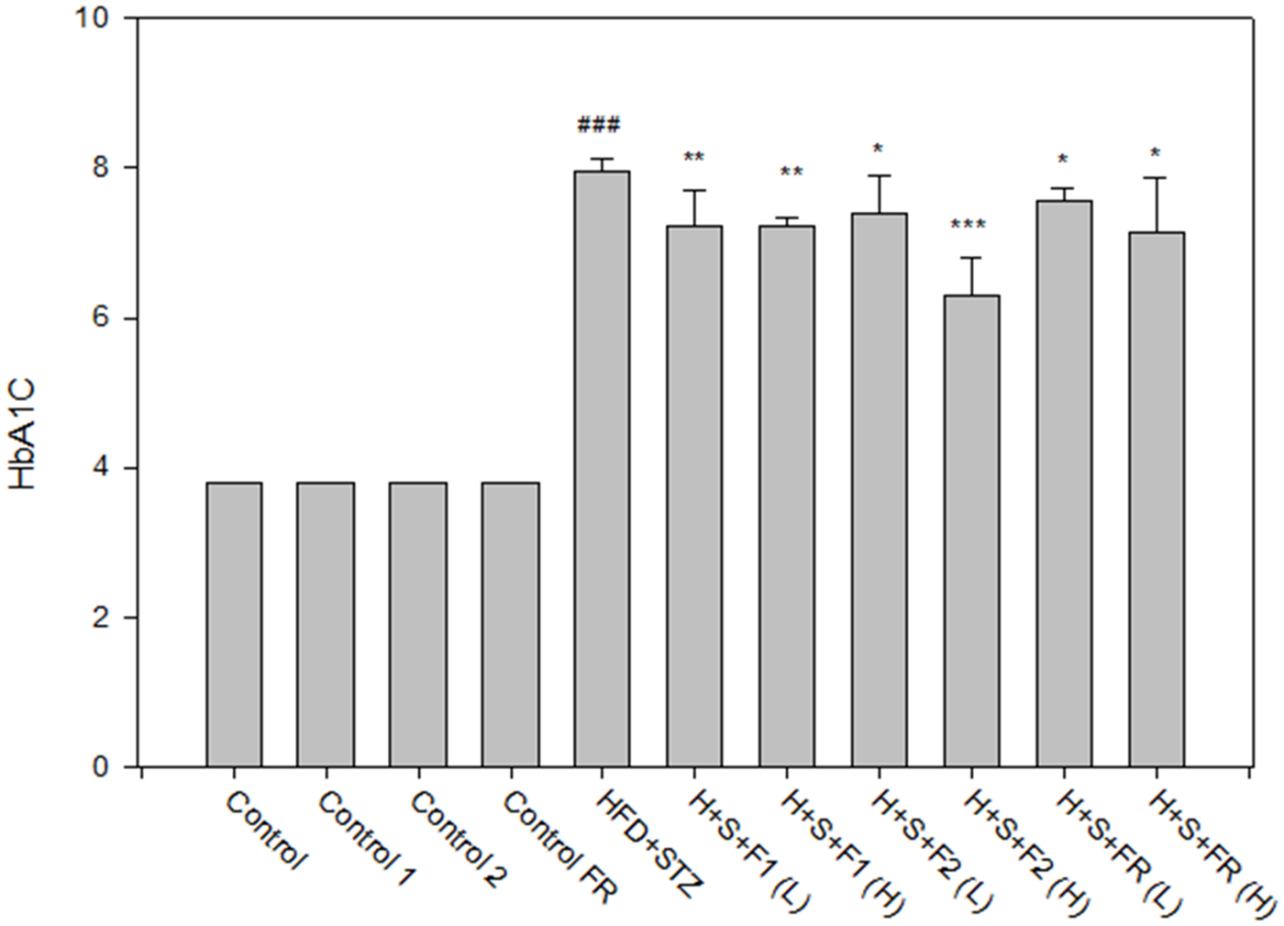
Effect of AE on HbA1C. Serum from the experimental animals was collected after sacrifice. The percentage of serum HbA1C was estimated by agglutination assay. Data are presented as mean±SD (n = 8 per group), and analyzed with ANOVA and post- test. ###*p* < 0.001, compared with the controls; *p < 0.05, **p < 0.01, ***p < 0.05, compared with HFD+STZ.

At the end of the experiments, HbA1C had doubled in the untreated diabetic group. All of the AE subfractions significantly decreased HbA1C, especially F2 (H), which reduced HbA1C from 8.0% to 6.5% compared to 3.9% in the controls. F1 (H) also showed good potency, reducing HbA1C from 8.0% to 7.3% (Fig 3).

### AE subfractions improved dyslipidemia accompanied with diabetes

**Fig 4:**
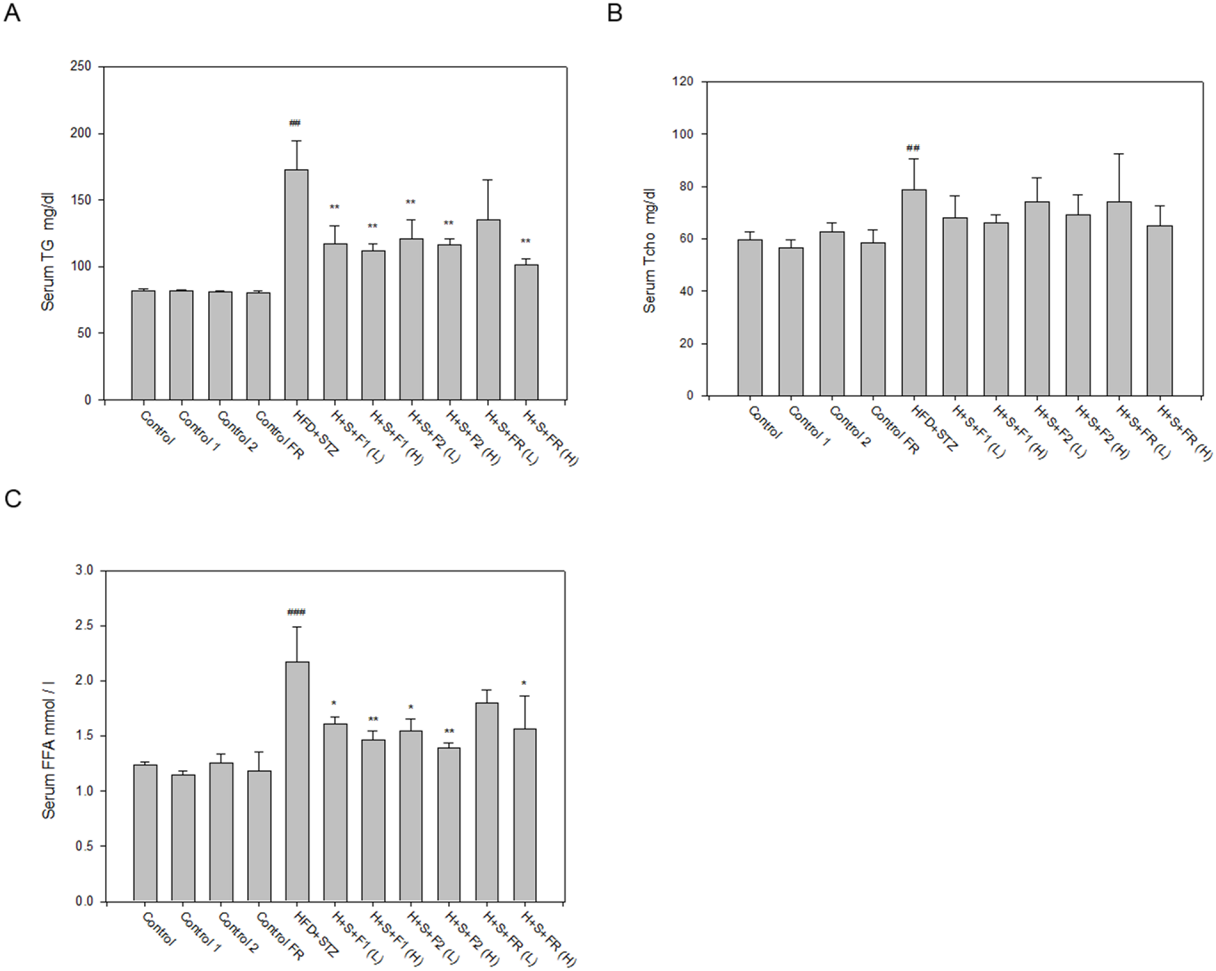
Effect of AE on dyslipidemia. Serum from the experimental animals was collected after sacrifice. The markers of lipid parameters were analyzed using enzymatic colorimetric methods. (A) serum TG; (B) serum cholesterol; (C) serum FFA. Data are presented as mean±SD (n = 8 per group), and analyzed with ANOVA and post- test. ##*p* < 0.01, ###*p* < 0.001, compared with the controls; *p < 0.05, **p < 0.01, compared with HFD+STZ.

The serum level of TG more than doubled in the untreated diabetic group. All of the AE subfractions effectively lowered the level of TG (Fig 4A). Serum cholesterol was also elevated in the diabetic group. However, total cholesterol was not altered by AE treatment (Fig 4B).

FFA is an important morbidity factor associated with obesity and metabolic syndrome. In the untreated diabetic group, FFA was increased by nearly 80%. AE treatment decreased the level of FFA in a dose-dependent manner (Fig 4C). These data suggested that all of the AE subfractions possessed a good ability to improve dyslipidemia in the diabetic rats.

### AE subfractions improved the profile of lipoproteins

**Fig 5:**
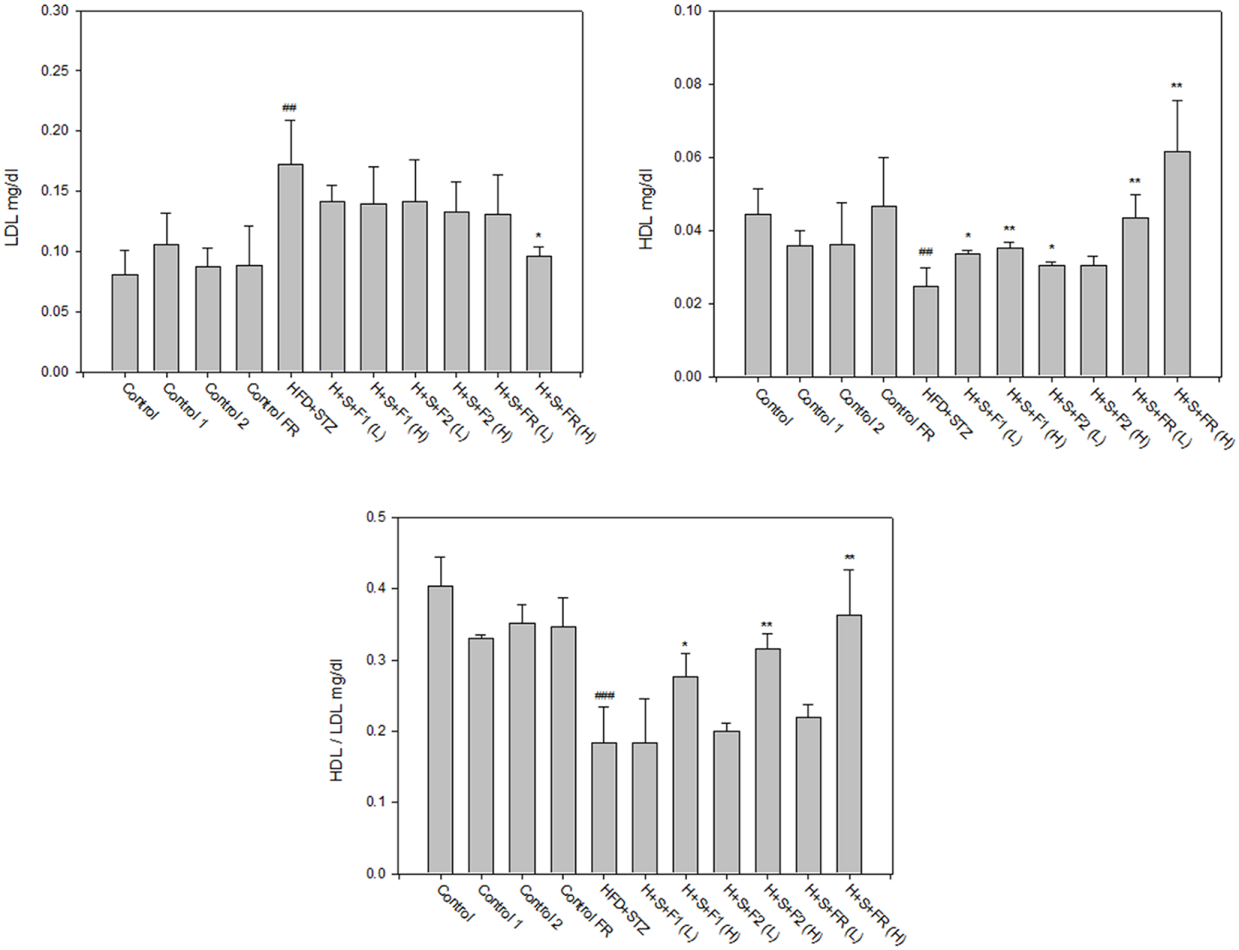
Effect of AE on lipoproteins. Serum from the experimental animals was collected after sacrifice. Lipoproteins were analyzed using enzymatic colorimetric methods and calculated as LDL, HDL, and HDL/LDL. Data are presented as mean±SD (n = 8 per group), and analyzed with ANOVA and post- test. ##*p* < 0.01, ###*p* < 0.001, compared with the controls; *p < 0.05, **p < 0.01, compared with HFD+STZ.

AE subfractions seemed to attenuate the high level of LDL accompanied with diabetes, although the effect was not statistically significant. HDL was decreased by nearly half in the diabetic group, but was elevated with AE treatment and especially with FR, which increased the level in a dose-dependent manner. The ratio of HDL/LDL revealed that all of the AE subfractions were beneficial for the distribution of lipoproteins (Fig 5).

### AE subfractions balance the diabetic weight change

**Fig 6:**
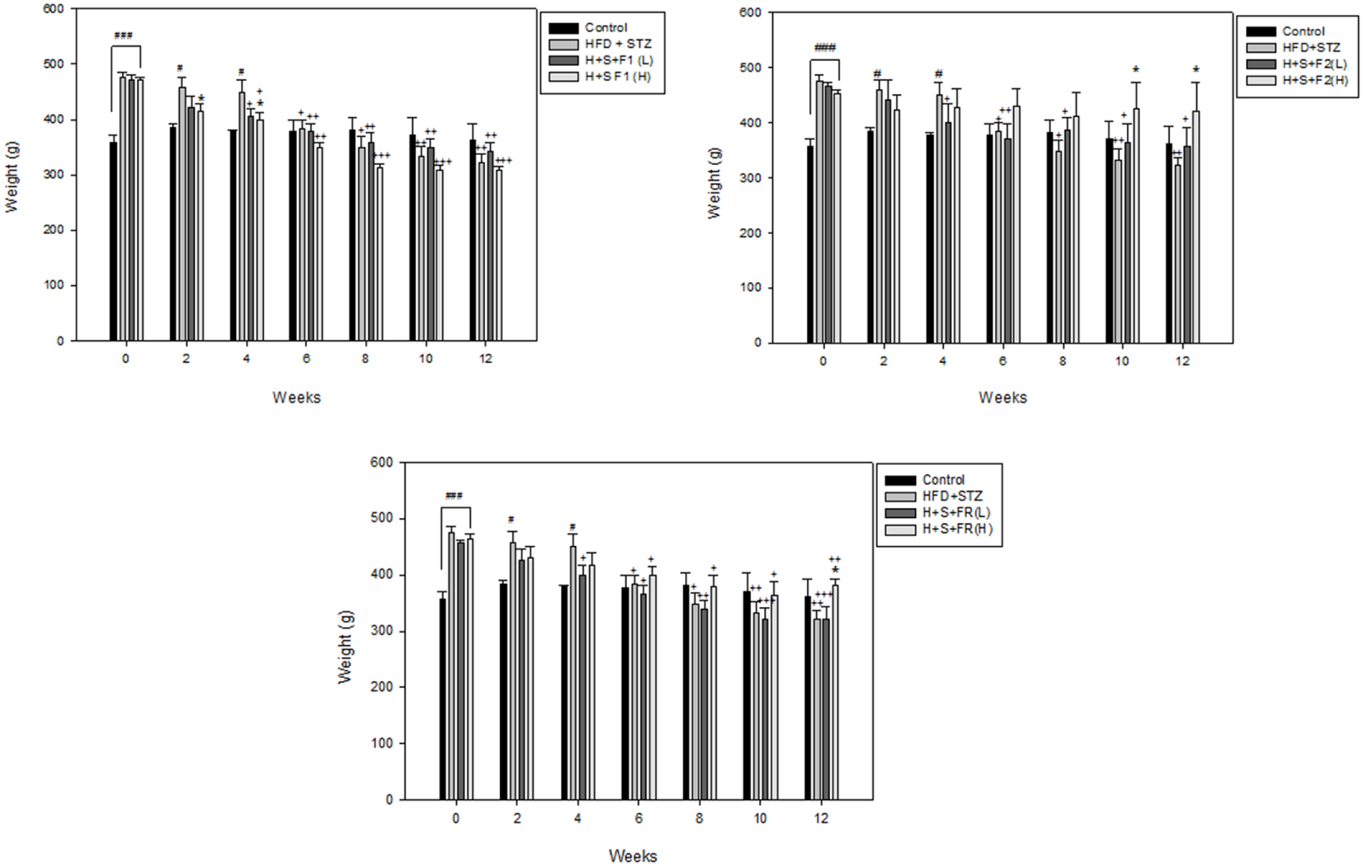
Effect of AE on body weight change. Rats were fed with different diets and weighed every week until the end of the experiment. Data are presented as mean±SD (n = 8 per group), and analyzed with ANOVA and post- test. #*p* < 0.05, ###*p* < 0.001, compared with the controls; *p < 0.05, compared with HFD+STZ; + *p* < 0.05, ++*p* < 0.01, +++*p* < 0.001, compared with the same group at 0 week.

All of the diabetic rats were much fatter at baseline. However, at 6 weeks, the untreated diabetic group lost weight and continued to lose weight until the end of the study. F2 (H) treatment significantly recovered the weight to even the baseline at 0 weeks, whereas F1 (H) reduced the weight from 2 weeks and then continued this effect until 12 weeks. These results implied the different modulation effects among the AE subfractions (Fig 6).

### AE was safe for the liver and kidneys

**Fig 7:**
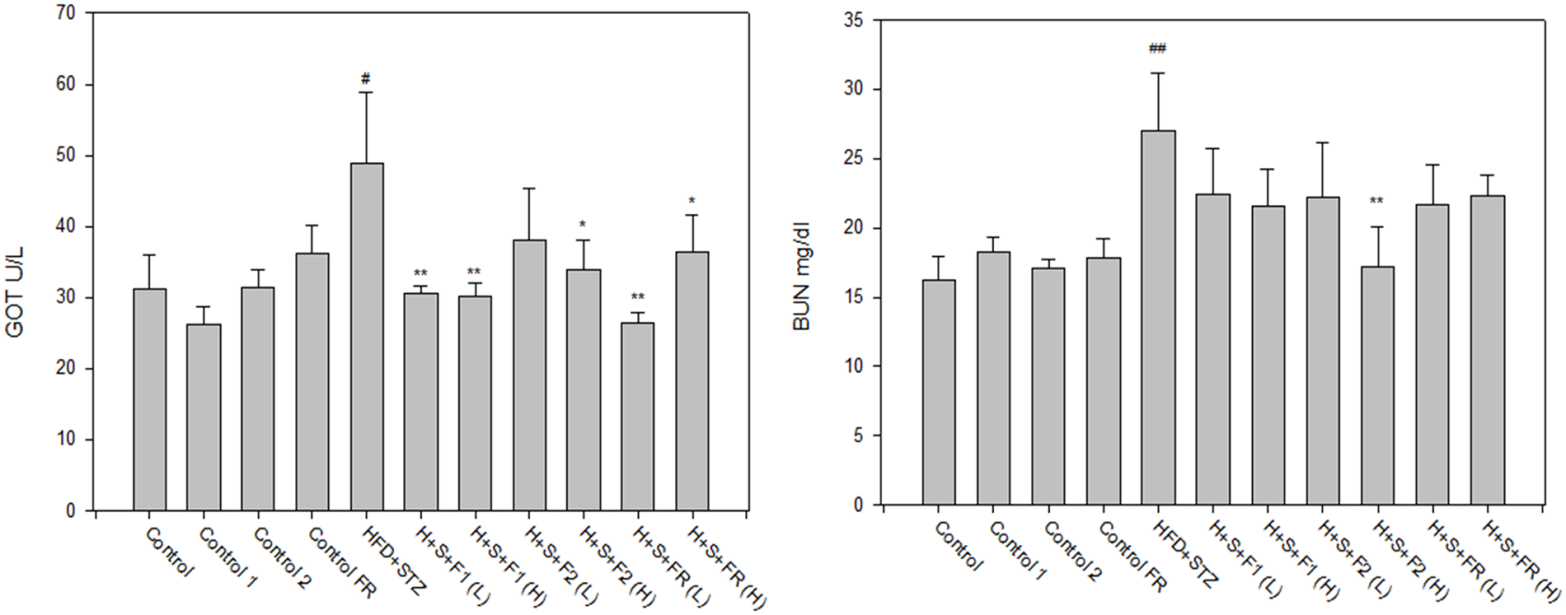
Safety evaluation of AE. Serum from the experimental animals was collected after sacrifice. GOT and BUN were measured as indicators detecting liver and kidney function, respectively.

Safety evaluation revealed that AE treatment alone did not harm the liver and kidneys. F2 (H) even returned the diabetes-increased BUN to the level of the controls. In addition, treatment with all of the AE subfractions significantly returned the level of GOT to the level of the controls, suggesting the liver-protective ability of AE (Fig 7).

## Discussion

In the present study, we used a type 2 diabetic model to test the effects of all AE subfractions in vivo. F2, which is rich in polysaccharides and carbohydrates, most strongly attenuated hyperglycemia, insulin resistance and HbA1C. Moreover, F2 showed a potent effect to improve dyslipidemia and prevent body weight loss accompanied with metabolic disturbances.

A few recent reports have suggested that polysaccharides may be able to improve metabolic disorders. The Ganoderma atrum polysaccharide was shown to have the potential to treat hyperglycemia, hyperlipidemia, and hyperinsulinemia in type 2 diabetic rats [11]. Furthermore, it has been shown to ameliorate hyperglycemia-induced endothelial cell death by inhibiting oxidative stress and subsequent mitochondrial dysfunction [12]. A Rehmannia glutinosa polysaccharide has been shown to effectively ameliorate hyperglycemia, hyperlipidemia, vascular inflammation and oxidative stress in STZ-induced diabetic mice, and thus it could be a potential therapeutic option for type 1 diabetes [13]. In addition, a Grifola frondosa polysaccharide has been shown to activate the insulin receptor protein, increase the metabolism of glucose, and stimulate synthesis of intracellular glycogen through the Akt/GSK-3 pathway in HepG2 cells [14]. Moreover, an Astragalus polysaccharide has been shown to improve palmitate-induced insulin resistance by inhibiting PTP1B and NF-kappaB in C2C12 myotubes [15]. The chemical analysis in our previous study revealed that F2 contains various monosaccharides or derivatives, among which myoinositol and rhamnose have been reported to be anti-hyperglycemic or anti-glycative [16, 17]. A clinical trial revealed that myo-inositol supplementation significantly decreased fasting glucose, insulin, HOMA-IR, and increased adiponectin [16]. An in vitro experiment demonstrated that at relatively low concentrations (between 10 and 100 μg ml−1), several rhamnose-rich oligo- or polysaccharides provided significant protection against cytotoxicity induced by advanced glycation end product [17]. As a glycation product of hemoglobin, an HbA1C level less than 7.0% is the treatment goal for diabetes [18]. We demonstrated a reduction in HbA1C to 6.5% with F2 (H) treatment. Hence the superior effect of F2 to attenuate hyperglycemia, insulin resistance and HbA1C could be attributed to the composition of polysaccharides and carbohydrates.

Our data revealed increased serum levels of TG, FFA, and ratio of HDL/LDL in the diabetic groups, showing the complex outcomes of metabolic disturbance led by insulin resistance. FFA is considered to play a critical role in metabolic complications such as dyslipidemia and insulin resistance. FFA-associated insulin resistance has been reported to be mediated by a molecular mechanism involving GPR40 and insulin receptor substrate-1 serine kinase, which play critical roles in developing insulin resistance and regulating the insulin receptor signaling pathway, respectively [19]. Such flux of FFA toward liver results in an increase in TG deposition and secretion of TG-rich lipoproteins, which in turn affects lipoprotein lipase (LPL) activity and the distribution of lipoprotein subtypes (1). Regulation of the adipose tissue LPL is significantly affected in individuals with insulin resistance in the postprandial period, and the presumed impairment could contribute to atherogenic dyslipidemia [20]. Moreover, a lack of LPL in skeletal muscles has been reported to result in insulin resistance in other key metabolic tissues [21], while increased LPL activity has been reported to improve insulin resistance and reduce adipose accumulation in transgenic rabbits [22]. All of the AE subfractions reduced markers of dyslipidemia such as TG and FFA. Among them, FR recovered HDL/LDL to the level of the controls and is considered to be the most advantageous to improve the lipoprotein profile.

Although type 2 diabetes mellitus is associated with obesity, in our diabetes model it was notable that the diabetic rats lost weight after a few weeks. This phenomenon coincides with a common clinical finding among patients with type 2 diabetic, and represents an imbalance in energy expenditure caused by impaired insulin function. However, with F2 and FR treatment, the experimental animals regained weight, implying a recovery of metabolic balance. In contrast, the rats that received F1 treatment continued to lose weight while still showing beneficial effects in all of the serum markers. Our previous analysis showed that F1 is rich in flavonoid and quercetin glycosides. Quercetin has been reported to regulate AMPK and MAPK signaling, and thus exert an anti-obese effect [23]. In addition to flavonoids, many anthocyanins are found in okra. Recently, oligomeric proanthocyanidins from *Abelmoschus esculentus* was reported to be the active compound inhibiting α-amylase and α-glucosidase. In addition to improving hyperglycemia, a reduction in starch digestion may contribute to weight loss [24].

In conclusion, AE shows potential to improve diabetes and its associated metabolic disturbances. The different composition and pathophysiologic modulation of individual subfractions should be considered during application and development of AE products. Further studies are warranted to investigate the role of AE as adjuvant therapy for diabetes.

## Supporting information

S1 Table Identified compounds in F1. (DOC)

S2 Table Monosaccharides in F2. (DOC)

S3 Table Diet for the animal experiment. (DOC)

S1 Fig AE prevents the exacerbation of β islet. (DOC)
