# Supplementary material for: The nutraceutical benefits of subfractions of Abelmoschus esculentus in treating type 2 diabetes mellitus
Source: PLoS One. 2017 Dec 7;12(12):e0189065. doi: 10.1371/journal.pone.0189065 (PMC5720626; doi:10.1371/journal.pone.0189065)
Supplement: S3 Table — (DOC) [file pone.0189065.s003.doc]

**S3. Diet for the animal experiment (C: control; H: high fat-diet)**

| Ingredient (%) | C | H |  |
| --- | --- | --- | --- |
| Casein | 20.0 | 20.0 |  |
| Sucrose | 6 | 6 |  |
| Corn starch | 51.8 | 37.8 |  |
| Corn oil | 12.0 | 25.9 |  |
| Cholesterol |  | 0.1 |  |
| Mineral premix | 4 | 4 |  |
| Vitamin premix | 1 | 1 |  |
| Choline | 0.2 | 0.2 | |
| Cellulose | 5 | 5 | |
| Total | 100 | 100 | |
